# Craniocervical Posture and Malocclusion: A Comprehensive Literature Review of Interdisciplinary Insights and Implications

**DOI:** 10.3390/medicina60122106

**Published:** 2024-12-22

**Authors:** Andreea Kui, Alexandru Bereanu, Ana-Maria Condor, Dalia Pop, Smaranda Buduru, Anca Labunet, Sebastian Șoicu, Rareș Buduru, Andrea Chisnoiu

**Affiliations:** 1 Prosthetic Dentistry Discipline, Department 4—Prosthetic Dentistry and Dental Materials Department, “Iuliu Hatieganu” Medicine and Pharmacy University, 400006 Cluj-Napoca, Romania; gulie.andreea@umfcluj.ro (A.K.); dana.buduru@umfcluj.ro (S.B.);; 2 Faculty of Dental Medicine, “Iuliu Hatieganu” Medicine and Pharmacy University, 400012 Cluj-Napoca, Romania; alexandru.bereanu@yahoo.com; 3 Oral Health Discipline, Department 3—Oral Rehabilitation, Faculty of Dental Medicine, “Iuliu Hatieganu” University of Medicine and Pharmacy, 400012 Cluj-Napoca, Romania; 4 Cluj County Emergency Clinical Hospital, 400006 Cluj-Napoca, Romania; 5 Dental Materials Discipline, Department 4—Prosthetic Dentistry and Dental Materials Department, “Iuliu Hatieganu” Medicine and Pharmacy University, 400089 Cluj-Napoca, Romania; 6 Dental House Pitești Private Practice, Argeș, 110062 Pitești, Romania; sebsoicu@yahoo.com; 7 Stomestet Private Practice, 400372 Cluj-Napoca, Romania

**Keywords:** craniocervical posture, malocclusion, orthodontic treatment

## Abstract

*Background and Objectives*: The impact of craniocervical posture on malocclusion has long intrigued researchers in dentistry, orthodontics, and physical therapy. This research aims to elucidate the relationship between craniocervical posture and both dental and skeletal malocclusions and to explore the potential for integrated multidisciplinary therapeutic approaches. *Materials and Methods*: We analyzed peer-reviewed articles published between 2013 and 2023 from PubMed/Medline, Web of Science, EMBASE, and Scopus. The search strategy included terms related to craniocervical posture and malocclusion, focusing on studies that evaluated the relationship between these conditions before and after various orthodontic or surgical treatments. *Results*: A total of 20 studies met the inclusion criteria, providing nuanced insights into the interplay between malocclusion types and craniocervical alignment. Findings suggest that altered craniocervical posture is more prevalent in individuals with skeletal malocclusions. In particular, orthodontic treatment and orthognathic surgery appear to influence craniocervical posture, suggesting a bidirectional relationship between craniofacial structure and neck alignment. *Conclusions*: Our literature review confirms a significant association between craniocervical posture and malocclusion, emphasizing the need for an integrative approach to the diagnosis and treatment of craniofacial anomalies. Future research should aim to quantify these relationships further through longitudinal studies, thereby increasing the understanding necessary to develop comprehensive treatment protocols.

## 1. Introduction

Craniocervical posture represents the biomechanical balance between the cervical spine and the cranium. While ideal posture is sought, most individuals exhibit suboptimal craniocervical alignment, with forward head posture being a common problem observed in the sagittal plane [1]. Several factors can disrupt normal head and neck alignment, including compromised vestibular, visual, and proprioceptive inputs, changes in vertebral morphology, alterations in neuromuscular function, and degeneration of arthritic facet joints [2]. In addition, a forward head position that compromises craniocervical posture has been found to relieve myofascial pain in the cervical muscles. The normal aging process contributes to this problem through degenerative changes in vertebral structures, leading to the narrowing of intervertebral disc spaces, reduced neck mobility, decreased strength in cervical and masticatory muscles, and ultimately poorer postural stability [1,3].

Poor craniocervical posture, especially forward head posture, has multiple negative effects on overall health. This posture can impair respiratory function by weakening the muscles involved in breathing [4]. It can also limit the range of motion of the cervical spine due to compromised cervical spine posture [5]. In addition, the temporomandibular joints may suffer, along with increased stiffness in the masticatory muscles. Such changes in head and neck position can affect the function of the masticatory muscles and jaw movements [1,6,7].

The complex relationship between craniocervical posture and malocclusion has received significant attention in dentistry, orthodontics, and physical therapy. Malocclusion, defined as the misalignment of teeth and jaws, not only affects dentomaxillary functions but also has implications for the musculoskeletal system [8]. The craniocervical posture, which indicates the neuromuscular balance between the cervical spine and the skull [1], can be compromised by poor alignments, such as forward head posture, resulting in muscular imbalances [9]. This anatomical proximity has led to the hypothesis of a possible relationship between craniocervical posture and malocclusion.

The relationship between craniocervical posture and malocclusion has been studied for almost a century, and despite promising results from various studies, the hypothesis that there is a relationship between craniocervical posture and malocclusion continues to be questioned by some authors [10,11,12,13,14,15]. In 1992, Martensmeier et al. observed modifications of cervical spine curvature in the sagittal plane following orthodontic treatment for malocclusion [12]. The research conducted by Tardieu et al. analyzed the influence of a dental occlusion perturbation on postural control. Their results have identified a direct link between dental occlusion and postural control in dynamic conditions and in the absence of visual signals [13].

However, in contrast to the aforementioned studies, other research in literature documented the absence of a correlation between craniocervical posture (equilibrium with closed and open eyes, loading distribution on feet, body oscillations) and dental occlusion [10,14].

Given the importance of malocclusion to dental students, dental practitioners, and researchers, there is a need for further understanding of its causes and mechanisms. Investigating the complex relationship between malocclusion and craniocervical posture is critical and suggests a potential multidisciplinary strategy involving physiotherapists in the management and prevention of malocclusion.

In this context, the purpose of this study is to review recent literature on craniocervical posture and malocclusion to determine if there is an association between the two. The aims of our study were as follows: to determine whether there is an association between altered craniocervical posture and a higher prevalence of malocclusions; to assess whether changes in craniocervical posture are more common in patients with skeletal malocclusions; to investigate whether orthodontic treatment of malocclusions affects craniocervical posture; to investigate whether orthognathic surgery for malocclusions affects craniocervical posture.

## 2. Materials and Methods

### 2.1. Search Strategy

We conducted a comprehensive search in the electronic databases Pubmed/Medline, Web of Science, EMBASE, and Scopus. The search had no time constraint and concluded on 2 December 2023. The search strategy included the utilization of the following terms and keywords: “craniocervical”, “head”, “neck”, “malocclusion”, “dental”, “skeletal”, “jaw”, “mandible”, and “posture”. In Table 1, the search strategy tailored for each database is displayed in its entirety. This literature review was conducted in accordance with the guidelines provided by the “Preferred Reporting Items for Systematic Reviews and Meta-Analyses (PRISMA) 2020 Checklist” [16].

### 2.2. Eligibility Criteria

The search terms and keywords that were used to determine the inclusion and exclusion criteria and the search strategy were obtained based on the formulation of a research question following the PICOTS acronym. The research question was, “In both adult and pediatric patients, does malocclusion, compared to functional occlusion, influence the craniocervical posture?”

- P (Population) = Adult and Pediatric Patients;
- I (Investigated condition) = Malocclusion;
- C (Control) = Functional Occlusion;
- O (Outcome) = Altered Craniocervical posture;
- T (Time) = Articles published between 2013–2023;
- S (Study design) = Randomized clinical trials, cohort, case reports, cross-sectional prospective and retrospective study designs.

Inclusion criteria were as follows: studies that were randomized clinical trials, cohort studies, case reports, cross-sectional prospective or retrospective in design; articles with full-text access; articles published in English; articles published between 2013 and 2023; studies involving human subjects; studies evaluating the relationship between craniocervical posture and malocclusion, before treatment and/or before and after treatment.

Exclusion criteria included articles not relevant to the topic under review, narrative reviews, scoping reviews, systematic reviews, meta-analyses, and any other publication that did not meet the inclusion criteria.

### 2.3. Selection Process

The selection process was performed by two independent researchers (A.K. and A.B.). The results of the four database searches were saved in the Zotero application [17]. The duplicate articles were removed manually within the software. Afterward, each publication was screened based on its title and abstract for their significance regarding the topic of interest. Full-text access was obtained for each article that had passed the screening; if the full text could not be acquired, the article was dismissed and deemed as not being retrieved. The articles that had passed the initial screening and had their full text accessible entered the phase of assessing their eligibility based on their title and abstract according to the inclusion and exclusion criteria. In the case where the abstract could not provide sufficient data to decide regarding its inclusion or exclusion, the article’s full text was examined by a third researcher (AMC) for a more thorough review.

### 2.4. Data Collection Process

Data for this study were systematically collected using Microsoft Excel (version 16.50), with rigorous checks to ensure completeness and accuracy during the extraction process.

The data extracted for each study included (1) Title and year of publication, (2) Objective of the study, (3) Study design, (4) Sample size and their ages, (5) The craniocervical posture analysis method, (6) Results and conclusion, (7) Type of orthodontic treatment, (8) Duration of the orthodontic treatment, and (9) Type of orthognathic surgery.

### 2.5. Critical Appraisal of Included Studies

Although no specific bias assessment scale was used in this review, a comprehensive bias evaluation was embedded in the study selection and data extraction processes to help minimize potential bias and enhance the credibility of the findings. Each study included in the review was assessed for bias in several key areas, including (1) study design and execution: attention was given to how each study’s design might predispose it to selection, implementation, detection, and attrition bias; (2) population and sampling methods: the selection criteria and sampling methods of each study were evaluated to determine the risk of selection bias and its potential impact on the generalizability of the results; (3) measurement consistency: the consistency and reliability of outcome measures across studies were assessed to identify any measurement bias that could influence the reported results.

## 3. Results

### 3.1. Study Selection

**Figure 1:**
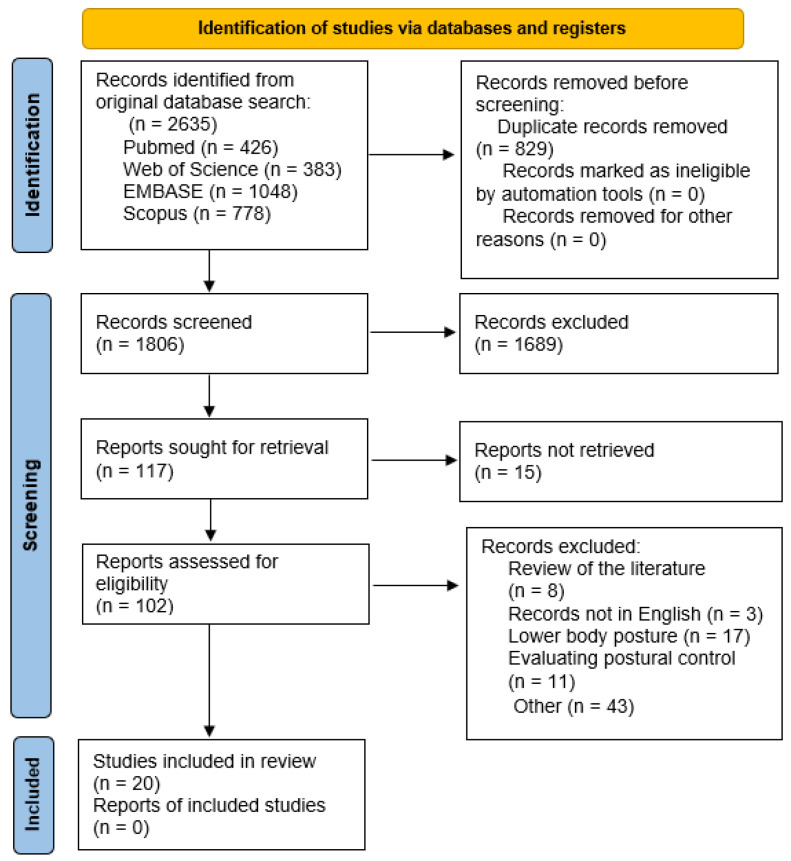
PRISMA flow diagram.

A total of 2635 records were found after applying the search strategy within the four databases: PubMed, Web of Science, EMBASE, and Scopus. After removing 829 duplicate records, the following 1806 records were screened, of which 1689 records were excluded. The remaining 117 records were sought for retrieval, where 15 were not retrieved. The 102 records remaining were assessed for their eligibility according to the established inclusion and exclusion criteria. Out of the records assessed for eligibility, 82 were excluded. A total of 20 studies were included in the qualitative synthesis. A PRISMA flow diagram was used to depict the identification, screening, and inclusion process (Figure 1).

One article investigated the relationship between craniocervical posture and dental malocclusion. Eight articles evaluated the relationship between craniocervical posture and skeletal malocclusion, all of them being observational, cross-sectional studies in design. Six articles evaluated the relationship between craniocervical posture and the treatment of malocclusion following orthodontic therapy. Five articles evaluated the relationship between craniocervical posture and the treatment of malocclusion following orthognathic surgery, all of them being cohort studies in design.

### 3.2. Craniocervical Posture and Malocclusion

Initially, we reviewed studies investigating the relationship between malocclusion and overall posture. Through this process, we identified nine relevant articles that explore this connection. These articles are summarized in Table 2.

### 3.3. Craniocervical Posture and Orthodontic Treatment

Six scholarly articles were identified, examining the complex relationships between craniocervical posture and orthodontic treatment. These studies explored the intricate connections and potential correlations within the biomechanical dynamics of the craniofacial complex, cervical morphology, and head alignment in the context of orthodontic therapies, as outlined in Table 3.

### 3.4. Craniocervical Posture in the Context of Orthognathic Surgery

This section analyzes recent scientific studies that investigate the connections between dental malocclusion, foot posture, and body balance in pediatric populations, highlighting potential correlations and their implications for postural health. A summary of these studies is provided in Table 4.

## 4. Discussion

A reduction in jaw size as a consequence of evolutionary processes has frequently been identified as a contributory factor in the elevated prevalence of dental malocclusion [38]. Given the increased prevalence of dental malocclusion, there has been relatively little exploration of the correlation between craniocervical posture and dental malocclusion. This review identified only one article that evaluated the relationship between craniocervical posture and dental malocclusion. Uysal et al. [18] investigated the relationship between the natural head posture and the crowding of the lower incisors. Their findings indicated a statistically significant positive correlation between head flexion in the natural head position and lower incisor crowding in male subjects.

Other authors, such as Pachì et al. [39], have also identified a statistically significant correlation between lower arch crowding and craniocervical posture. However, their findings indicate that an increased head extension in the natural head position was associated with an increased prevalence of lower arch crowding. The findings of Pachì et al. [39] are in accordance with the “soft-tissue stretching” theory, which posits that the extension of the head can result in dorsal-caudal pressure being exerted on both the teeth and the bones of the face due to the stretching of the soft tissue layers [31]. This pressure has the potential to disrupt the alignment of the dental arch.

While the “soft tissue stretching” theory proposed by Solow and Kreiborg [40] appears to offer a promising explanation for the potential relationship between craniocervical posture and dental malocclusion, it fails to provide an explanation for the observed relationship in the study included in this review (Uysal et al. [35]). Consequently, it can be inferred that an alternative mechanism must be responsible for this observed relationship. One potential explanation is Proffit’s ‘Equilibrium’ theory, which posits that the teeth are subject to continuous pressure from the tongue, lips, and cheeks. For the correct alignment of the teeth, a balance must be achieved between the internal (tongue) and external (lips and cheek) forces. A disruption of this muscular balance will result in dental malocclusion. Such a muscular imbalance was described by Hellsing and L’Estrange [41] during the flexion of the head. However, this is insufficient to justify the prevalence of lower incisor crowding in patients with a flexed head posture, as observed by Uysal et al. [18].

This review identified a single study that investigated the relationship between craniocervical posture and dental malocclusion. The study found a statistically significant relationship. Nevertheless, the precise mechanism of this relationship remains unclear and inconsistent with the conclusions drawn from other published studies with similar research objectives. It is recommended that this study be improved by using a method of evaluating craniocervical posture that does not involve a device placed on the subject’s head and neck, as this may potentially affect the reliability of the results. Furthermore, increasing the sample size and examining the differences between subjects with an extended and flexed craniocervical posture and the prevalence of lower incisor crowding and other dental malocclusions would be beneficial. Further investigation into the relationship between craniocervical posture and dental malocclusion would be beneficial as it would enhance understanding of the impact of posture on dentoskeletal development and have clinical significance in the prevention and treatment of dental malocclusion.

It remains unclear precisely how the craniofacial complex and cervical spine are anatomically interconnected and what influence each has on the other. The review included eight articles that investigated the relationship between craniocervical posture and skeletal malocclusion.

The study designs of all eight articles were observational cross-sectional, which is regarded as one of the lowest-quality designs in evidence-based medicine due to its susceptibility to threats to internal validity [42]. All forms of skeletal malocclusion were investigated in the sagittal plane, with subjects classified according to their ANB angle into skeletal Classes I, II, and III. The sample sizes ranged from 45 subjects to 163 subjects, with a greater number of subjects included in studies evaluating younger individuals (i.e., those under 18 years of age). One potential reason is that the inclusion criteria for these studies require individuals who have not undergone treatment for their skeletal malocclusion. Consequently, there is a reduced chance of including an individual who has not received any treatment at an older age.

All eight articles used cephalometric analysis to evaluate the craniocervical posture. A variety of measurements were employed for the evaluation of cervical, craniofacial, and craniocervical postures. The most frequently occurring measurements were OPT/HOR, CVT/HOR, SN/OPT, SN/CVT, FH/CVT, and FH/OPT angles across the eight articles. However, it should be noted that not all the articles presented a common measurement for the craniocervical posture. However, the lack of consistency in the measurements of the craniocervical posture across studies may lead to difficulties in comparing the studies, as the inconsistency in measurements makes it challenging to draw reliable conclusions, and the researchers may find it challenging to interpret the findings.

Of the eight articles that evaluated the relationship between craniocervical posture and skeletal malocclusion, five identified a statistically significant relationship for at least one skeletal class. Four studies observed a statistically significant relationship between craniocervical posture and skeletal Class II malocclusion. In the articles that identified a statistically significant relationship, the subjects with skeletal Class II malocclusion exhibited greater head extension in their natural head position. This was also observed in the articles that identified a non-statistically significant relationship. The “soft tissue stretching” theory postulated by Solow and Kreiborg provides an explanation for this relationship. As the head extends, the resistance of the soft-tissue layers covering the head and neck exerts a dorsal-caudal force on the mandible, impeding its growth in the sagittal plane [40,43,44]. A reduction in the size of the upper airway can result in cephalic hyperextension, which is a reflex action that facilitates the passage of air. It has been demonstrated that children with enlarged tonsils and allergic rhinitis exhibit an increase in craniocervical angles. Vig et al. [45] demonstrated that when the nasal airway is obstructed, there is an immediate increase in the craniocervical angles. Upon removal of the obstruction, the head reverts to its usual posture.

In addition, three of the eight articles examined identified a statistically significant relationship between craniocervical posture and skeletal Class III malocclusion. The subjects with skeletal Class III malocclusion exhibited a more flexed head and forward head posture in the natural head position throughout the articles, which identified a statistically significant and a non-statistically significant relationship. Additionally, Solow and Tallgren observed that individuals with a more flexed head presented with prognathism of the jaws [43]. One possible explanation for this finding is the observation made by Hellsing and L’Estrange [41], who noted a reduction in perioral forces when the head was flexed, potentially leading to uncontrolled sagittal growth of the mandible.

Although the findings across the different articles do not consistently align regarding the precise association between skeletal Classes II/III and craniocervical posture, the majority have indicated a statistically significant correlation between craniocervical posture and skeletal malocclusion for at least one of the skeletal Classes. In reviewing the relationships between craniocervical posture and malocclusion, it was consistently observed that skeletal Class II malocclusions are often associated with an extended head posture, whereas Class III cases tend to correlate with a more flexed cervical posture. These findings suggest that an understanding of craniocervical dynamics is crucial for the effective management of malocclusions, potentially guiding more personalized treatment approaches. Future research should focus on longitudinal studies that can better track changes in posture over the course of orthodontic or surgical interventions and explore the biomechanical mechanisms underlying these changes.

### 4.1. Craniocervical Posture and Orthodontic Treatments

The stomatognathic system is closely related anatomically to the cervical spine. Changes in the mouth, jaws, and associated structures can affect craniocervical posture, demonstrating a morpho-functional relationship between the two systems. Six articles that evaluated the effects of orthodontic therapy in the treatment of malocclusion on craniocervical posture were included in this literature review.

All six articles were cohort types, which rank among the highest quality methodologies in evidence-based medicine, as they can establish a chronological link between exposure and outcome [42]. The sample sizes ranged from 12 subjects to 64 subjects. Five of the six articles included under 18 subjects in their study. The craniocervical posture method was mostly by means of cephalometric analysis of a lateral cephalogram (5 out of 6 articles); however, one article evaluated the head and neck posture by photogrammetric means using vertical laser lines. The photogrammetric analysis method is a suitable means of evaluating the head posture. Despite this, the cephalometric analysis seems to be the most indicated means of evaluating the craniocervical posture, as it allows for a greater objective perspective of the skeletal structure without the inter-reference of the overlying soft tissues [46].

Of the six articles, four evaluated the effects of functional orthodontic appliances, and two evaluated the effects of fixed orthodontic appliances on craniocervical posture. Of the six articles, four found a statistically significant effect of orthodontic appliances on craniocervical posture. There was a clear discrepancy between the achievement of a statistically significant effect on craniocervical posture and the type of orthodontic appliance used. In the articles evaluating the effects of functional appliances on craniocervical posture, a statistically significant effect was observed in 75% of the articles, whereas in the articles evaluating the effects of fixed appliances on craniocervical posture, a statistically significant effect was observed in only 50% of the articles. This may be explained by the effect of orthodontic treatment of malocclusion on surrounding muscle activity. Pittar et al. [47] observed that fixed orthodontic appliances reduced masticatory muscle activity compared to functional orthodontic appliances, which increased muscle activity. Stable dental occlusion achieved after successful orthodontic treatment also increases masticatory muscle activity [48]. Since there is strong evidence for a significant relationship between jaw and neck muscle activity [49], it can be explained how the changes in muscle activity of the jaw muscles after orthodontic therapy (especially functional orthodontic appliances) in the treatment of malocclusion can influence the cervical muscles and, in turn, affect head and neck posture.

Although promising results have been observed in this review describing a possible effect of orthodontic therapy in the treatment of malocclusion on craniocervical posture and subsequently demonstrating a relationship between craniocervical posture and malocclusion, further studies with a longer observation period to assess the stability of the results obtained are needed, The inclusion of control groups, a means of standardization in the analysis of craniocervical posture, and the evaluation of different types of orthodontic appliances should be undertaken in order to deepen our understanding of the effects of the treatments we perform as clinicians on our patients with malocclusion.

### 4.2. Craniocervical Posture and Orthognathic Surgery

Because of the abundant muscular attachments of the jaws, especially the mandible [9], changing the position of the jaws to correct malocclusion using orthognathic surgery can potentially affect the cervical muscles and spine, which in turn affect the craniocervical posture, providing evidence that there is a relationship between craniocervical posture and malocclusion. For this reason, the author included and reviewed five articles that examined the effects of orthognathic surgery in the treatment of malocclusion on craniocervical posture.

All six articles employed a cohort study design. Sample sizes ranged from 25 to 41 subjects. The method of craniocervical posture analysis varied among the five articles: cephalometric analysis (n = 3), photogrammetric analysis (n = 1), and cone beam computed tomography (n = 1). Several types of orthognathic surgery were performed between the studies, but they all had mandibular surgical correction in common. The mandible was either receded or advanced in conjunction with or without a Le Fort I osteotomy and genioplasty.

Of the five articles, three observed a statistically significant effect of orthognathic surgery in the treatment of malocclusion on craniocervical posture. There was no clear discrepancy between achieving a statistically significant effect on craniocervical posture and the type of orthognathic surgery performed. It was suggested that the associated Le Fort I osteotomies and genioplasties had little effect on changing craniocervical posture [35]. Therefore, the change in the position of the mandible was considered to be the main effect on the craniocervical posture.

However, it was observed that the effects on head and neck posture, regardless of whether the mandibular correction involved setback or advancement, were comparable to the patterns observed in the subjects with skeletal malocclusion. The subjects who underwent mandibular setback (posterior positioning of the mandible) presented with greater posterior head and neck tilt postoperatively compared to their preoperative head and neck position, similar to the findings of posterior head and neck tilt observed in the subjects with Class II skeletal malocclusion (posterior positioning of the mandible). Similarly, the subjects who underwent mandibular advancement (anterior positioning of the mandible) presented with greater anterior head and neck tilting postoperatively compared to their head and neck position preoperatively, with similar findings of anterior head and neck tilting observed in the subjects with skeletal class III malocclusion (anterior positioning of the mandible).

The explanation for the posterior tilt of the head after mandibular set-back is similar to the explanation for the relationship between craniocervical posture and skeletal class II malocclusion. It has been shown that after mandibular setback, pharyngeal airway space is reduced [50], and as a reflex to the upper airway obstruction, the head extends to facilitate breathing [44,45,46]. An alternative explanation may involve a disturbance in the neuromuscular balance of the head and neck. The posterior neck muscles, consisting of the posterior cervical extensors, counterbalance the anterior neck muscle groups, consisting of the platysma, suprahyoid, and infrahyoid muscles [1,51,52]. Valk et al. observed that after mandibular set-back surgery, the space from the mention to the hyoid decreased, and as a result, a reduction in both tension and length of the supra- and infrahyoid muscles was noted [50]. Therefore, the ability to create a new balance between the cervical muscles may explain the posterior tilting of the head in subjects after mandibular set-back surgery in the treatment of malocclusion.

A plausible explanation for the observed anterior tilting of the head in subjects after mandibular advancement surgery is described by Lin and Edwards [35]. Before surgery, the cervical muscles balance the patient’s head with the malocclusion in a natural head position. After surgery, when the mandible is advanced, the muscular attachments, such as the tongue and floor of the mouth, also move anteriorly, with the center of the head also moving anteriorly to maintain the neuromuscular balance of the head. In both cases, achieving a greater number of stable and functional occlusal contacts by correcting the malocclusion following orthognathic surgery may also affect the surrounding jaw musculature, with possible effects on the cervical muscles and subsequent changes in head and neck posture [48,49]. Based on these findings, the authors recommend that further studies be conducted to evaluate the relationship between craniocervical posture and the treatment of malocclusion after orthognathic surgery to investigate both the effects of orthognathic surgery and the relationship between the stomatognathic system and craniocervical posture. It should be noted that this review research is subject to a number of limitations. The inclusion of only English-language articles may introduce language bias, potentially excluding valuable studies published in other languages. This restriction could limit the comprehensiveness of the review by overlooking findings from non-English studies, which might offer unique insights or diverse perspectives, particularly if conducted in regions where English is not the primary language.

Orthognathic surgery, including procedures such as mandibular advancement or setback, typically results in significant postural adjustments. This review has found that such surgical changes have altered skeletal relationships, which subsequently affect the muscular and soft tissue structures of the neck and head. For example, mandibular setbacks may result in compensatory neck extension as patients naturally adjust their posture to maintain functional occlusion and optimal visual alignment. These adjustments are critical because they can significantly affect the patient’s overall posture.

### 4.3. Limitations of This Study

The findings of this literature review are interpreted with an awareness of the biases associated with the study designs. While these designs are appropriate for exploratory purposes, they inherently limit causal inference due to potential confounding and bias: (1) selection bias—the included studies may have differed in their selection criteria, which could influence the observed relationships between craniocervical posture and malocclusion; (2) measurement bias—differences in methods used to assess craniocervical posture could lead to inconsistencies in how this variable is conceptualized and measured, affecting the synthesis of findings; (3) publication bias—only studies published in English were included, potentially overrepresenting studies with significant findings and excluding relevant data published in other languages or unpublished data.

One notable limitation observed across the studies included in this review is the inconsistency in screening for nasal passage problems. While some studies explicitly excluded participants with known nasal obstructions or allergies, which could impact craniocervical posture and dental malocclusion, other studies did not mention any such screening. This discrepancy raises concerns about the comparability of study populations and the generalizability of the findings. The lack of uniform screening for nasal passage issues may result in confounding biases that could influence the reported relationships between craniocervical posture and malocclusion outcomes. Future studies should standardize participant screening processes to include evaluations for nasal passage issues to ensure more reliable and comparable results across different research efforts.

The studies included in this review investigating the relationship between craniocervical posture and skeletal malocclusion are limited in several ways. Firstly, the observational cross-sectional studies included do not establish a chronological link between exposure and outcome. Secondly, there is considerable variation in the measurement angle used to assess craniocervical posture across studies. Thirdly, not all studies included a control group, and malocclusions were only investigated in the sagittal plane. To enhance the rigor of future studies, it would be beneficial to extend the longitudinal observation period, increase the sample size, and standardize the methodology for analyzing craniocervical posture.

The exclusion of non-English articles from this review was guided by several practical considerations. One reason was the potential for misinterpretation of complex clinical and biomechanical terminology during translation, which could compromise the consistency and reliability of the analysis. Additionally, the resources required for accurate translation and interpretation of non-English studies were beyond the scope of this review. Importantly, most high-impact, peer-reviewed journals publish articles in English, ensuring that the review incorporates widely accessible, high-quality studies. By focusing on English-language publications, we aimed to maintain a high standard of methodological rigor and reduce the risk of misinterpretation.

The findings of this study indicate a potential correlation between craniocervical posture and malocclusion. This conclusion is based on an analysis of articles that evaluated the craniocervical posture before and after the treatment of malocclusion. Nevertheless, it is important to acknowledge the limitations of this literature review. These include the absence of an assessment of potential bias in the included articles, the inclusion of only those articles published in English, the inability to access all articles following the screening process, and the small number of studies available for inclusion.

### 4.4. Clinical Relevance and Future Directions

Given the anatomical interrelationships between the craniofacial complex and the cervical spine, further investigation of how these relationships influence orthodontic and orthognathic outcomes is critical. This review supports the notion that orthodontic treatments, particularly functional appliances, may have a significant impact on craniocervical posture, necessitating a comprehensive approach to clinical assessment [47,48,49]. In addition, findings from studies of orthognathic surgery suggest that surgical correction of the jaw can result in significant postural adjustments, underscoring the importance of considering postural changes in surgical planning [35,50,51,52].

Nevertheless, the predominance of cross-sectional studies in the current body of literature limits the ability to establish causal relationships between craniocervical posture and malocclusion. While cross-sectional studies provide valuable insights into potential associations, they capture a single point in time, which prevents a clear understanding of how changes in one variable might lead to changes in another. In contrast, longitudinal studies follow participants over time, enabling researchers to observe temporal changes and make stronger inferences regarding causality. For example, by tracking changes in craniocervical posture before, during, and after orthodontic or orthognathic treatment, longitudinal studies could offer clearer evidence of how these treatments impact posture. As such, we emphasize the importance of future research employing longitudinal designs to better elucidate the causal relationships between craniocervical posture and malocclusion.

### 4.5. Recommendations for Clinical Practice

Orthodontists and surgeons should be aware of the potential postural changes associated with malocclusion treatment. A multidisciplinary approach involving orthodontists, surgeons, and possibly physiotherapists may improve patient outcomes by addressing both dental and postural health comprehensively. Further studies, particularly those using longitudinal designs and larger, more diverse sample sizes, are recommended to explore the long-term effects of malocclusion treatment on craniocervical posture. Standardization of posture analysis and the inclusion of control groups in future studies may provide more definitive findings and aid in the development of evidence-based treatment protocols.

## 5. Conclusions

The complexity of the stomatognathic system continues to attract considerable interest among dental clinicians, students, and researchers. In this review of the literature, the hypothesized relationship between craniocervical posture and malocclusion was critically examined due to their close anatomical interrelationships. Our primary objectives were to assess the prevalence of altered craniocervical posture in individuals with malocclusions and to evaluate the impact of orthodontic and orthognathic interventions on the adjustment of posture.

The main findings of this review are as follows: (a) prevalence of postural changes: There is a significant association between craniocervical posture and both dental and skeletal malocclusions. In particular, patients with Class II skeletal malocclusions often have extended head and neck postures, whereas patients with Class III malocclusions tend to have more neck flexion; (b) impact of treatments: Both orthodontic treatment and orthognathic surgery have been shown to have an effect on craniocervical posture. This confirms that interventions aimed at correcting malocclusions can lead to significant changes in neck alignment.

The potential causal mechanisms underlying the relationship between craniocervical posture and malocclusion can be explained by several physiological and biomechanical theories. A key perspective is the soft tissue stretching theory proposed by Solow and Kreiborg. This theory suggests that head extension stretches the soft tissues of the neck, exerting a dorsal-caudal force on the mandible. This force may influence the sagittal development of the mandible, potentially contributing to Class II malocclusions. Another perspective is the muscle imbalance theory, which suggests that changes in head posture, particularly forward head posture, disrupt the balance between flexor and extensor muscles in the neck. This imbalance may alter the distribution of forces across the craniofacial complex, affecting the position of the mandible. Finally, the equilibrium theory proposed by Proffit states that the position of the tongue, lips, and cheeks creates a balance of forces that influence tooth alignment. Changes in craniocervical posture can affect the position of the tongue and alter the balance of forces acting on the teeth, potentially leading to malocclusion.

Despite these findings, existing studies are often limited in quality and scope, and the current body of evidence lacks the robustness needed to definitively establish a cause-and-effect relationship. Misinterpretation of these correlations may lead to diagnostic and therapeutic inaccuracies. There is a clear need for further high-quality, longitudinal research, although the findings underscore the potential benefits of incorporating physiotherapeutic approaches into comprehensive malocclusion management. Such studies would help to clarify these relationships in a more definitive manner and to substantiate the clinical implications of these findings.

## Figures and Tables

**Table 1:** Search strategy for each database.

Pubmed/Medline
((“craniocervical”[All Fields] OR (“head”[MeSH Terms] OR “head”[All Fields]) OR (“neck”[MeSH Terms] OR “neck”[All Fields])) AND (“postural”[All Fields] OR “posturally”[All Fields] OR “posture”[MeSH Terms] OR “posture”[All Fields] OR “postures”[All Fields] OR “postured”[All Fields] OR “posturing”[All Fields]) AND (“malocclusal”[All Fields] OR “malocclusion”[MeSH Terms] OR”malocclusion”[All Fields] OR “malocclusions”[All Fields] OR “malocclu- sive”[All Fields] OR (“dental health services”[MeSH Terms] OR (“dental”[All Fields] AND “health”[All Fields] AND “services”[All Fields]) OR “dental health services”[All Fields] OR “dental”[All Fields] OR “dentally”[All Fields] OR “dentals”[All Fields]) OR (“jaw”[MeSH Terms] OR “jaw”[All Fields]))) AND (2013:2023[pdat])
Web of Science
(craniocervical OR head OR neck) AND (posture) AND (malocclusion OR dental OR jaw) (Topic) and 2013 or 2014 or 2015 or 2016 or 2017 or 2018 or 2019 or 2020 or 2021 or 2022 or 2023 (Publication Years)
EMBASE
(craniocervical OR ‘head’/exp OR head OR ‘neck’/exp OR neck) AND (‘pos- ture’/exp OR posture) AND (‘malocclusion’/exp OR malocclusion OR ‘den- tal’/exp OR dental OR ‘jaw’/exp OR jaw OR ‘mandible’/exp OR mandible) AND [2013–2023]/py
Scopus
TITLE-ABS-KEY ((craniocervical OR head OR neck) AND posture AND (malocclusion OR dental OR skeletal OR jaw OR mandible)) AND PUBYEAR > 2012 AND PUBYEAR < 2024

**Table 2:** Studies investigating craniocervical posture and malocclusion.

No.	Authors	Title Year	Objective	Study Design	Sample Size and Ages	Posture Analysis Method	Conclusion
1.	Uysal T, Yagci A, Ekizer A, Usumez S	Natural head position and lower incisor irregularity, Is there a relationship? [18] 2016	Assess the relationship between natural head position and lower incisor crowding.	Cohort	103 subjects, 51 males (mean age: 14.20 years) and 52 females (mean age: 15.02 years).	Inclinometer device and a portable information logger.	Significant relationship between lower incisor crowding and sagittal natural head position in male children.
2.	Alexa VT, Fratila AD, Szuhanek C, Jumanca D, Lalescu D, Galuscan A 2022	Cephalometric assessment regarding craniocervical posture in orthodontic patients [19]	Investigated characteristics of craniocervical morphology in patients grouped according to their ANB angle and according to their vertical growth pattern before orthodontic treatment.	Observational cross- sectional	45 subjects, between 25–30 years	Cephalometric analysis of lateral cephalogram in natural head position.	Class II subjects have a greater head extension, and Class III subjects have a greater head flexion and forward head posture.
3.	Hedayati Z, Paknahad M, Zorriasatine F 2013	Comparison of Natural Head Position in Different Anteroposterior Malocclusions [20]	Investigate the natural head position of the three skeletal classes of malocclusion.	Observational cross-sectional	102 subjects, between 15–19 years	Cephalometric analysis of lateral cephalogram in natural head position.	No statistically significant correlations were observed for Class II. Class III subjects have a greater head flexion and forward head posture.
4.	Bernal LV, Marin H, Herrera CP, Montoya C, Herrera YU	Craniocervical Posture in Children with Class I, II, and III Skeletal Relationships [21] 2017	Investigate the relationship between craniocervical posture and the sagittal skeletal classification in children.	Observational cross-sectional	107 subjects, between 6–11 years	Cephalometric analysis of lateral cephalogram in natural head position.	No significant differences in craniocervical posture were observed between children with different malocclusions.
5.	Kale, B. Buyukcavus, M	Effect of Craniofacial Growth Pattern on Head Posture [22] 2020	Investigate differences in craniocervical posture according to craniocervical growth pattern.	Observational, cross-sectional	163 subjects under the age of 17 years	Cephalometric analysis of lateral cephalogram in natural head position.	Class III malocclusion and hypodivergent vertical growth patterns exhibited the lowest craniocervical angle. Head posture remained consistent across subgroups categorized by different types of Malocclusions
6.	Sandoval C, Díaz A, Manríquez G.	Relationship between craniocervical posture and skeletal class: A statistical multivariate approach for studying Class II and Class III malocclusions [23] 2019	Investigate the relationship between head posture and skeletal class.	Observational cross-sectional	65 subjects over the age of 18 years	Cephalometric analysis of lateral cephalogram.	Class II presented a greater head extension than Class III. No correlation was observed for subjects with Class III.
7.	Baidas LF	Relationship between head posture and anterior–posterior skeletal patterns in a group of female patients [24] 2014	Investigate the relationship between craniocervical posture and the anteroposterior skeletal class in female adults.	Observational cross-sectional	75 subjects, between 18–25 years	Cephalometric analysis of lateral cephalogram in natural head position.	No statistically significant differences observed. Tendency for Class II patients to have a greater head extension, and Class III patients exhibit a greater forward head posture.
8.	Vukicevic V, Petrovic D	Relationship between head posture and parameters of sagittal position and length of jaws [25] 2016	Investigate the relationship between craniocervical posture and the jaw length and position in the sagittal plane.	Observational, cross-sectional	90 subjects, between 8–14 years	Cephalometric analysis of lateral cephalogram.	Class II individuals exhibit the greatest head extension. No statistically significant correlations were observed for Class III.
9.	Liu Y, Sun X, Chen Y, Hu M, Hou X, Liu C	Relationships of sagittal skeletal discrepancy, natural head position, and craniocervical posture in young Chinese children [26] 2016	Investigate the relationship between the skeletal discrepancy in the sagittal plane, natural head posture, and craniocervical posture in Chinese children with an average facial pattern in the vertical plane.	Observational, cross-sectional	90 subjects, between 11–14 years	Cephalometric analysis of lateral cephalogram in natural head position.	Class II subjects exhibit a greater extended head posture. Class III subjects exhibit a greater flexed head posture.

**Table 3:** Studies investigating craniocervical posture and orthodontic treatment.

No.	Authors	Title Year	Orthodontic Device	Study Design	Sample Size and Ages	Posture Analysis Method	Conclusion
1.	Bardellini E, Gulino MG, Fontana S, Amadori F, Febbrari M, Majorana A	Can the Treatment of Dental Malocclusions Affect the Posture in Children? [27]	Functional orthodontic appliance (Mouth Slow Balance device)	Cohort	60 subjects, between 9–12 years	Photographic, Vertical laser line	Correction of dental malocclusion contributes to the significant rebalancing of the head posture.
2.	Malik N, Fernandes BA, Ramamurthy PH, Anjum S, Prakash A, Sinha A	Cephalometric evaluation of the cervical spine posture following fixed functional therapy with Forsus appliance [28] 2022	Functional orthodontic appliance, (Forsus appliance)	Cohort	12 subjects, between 13–18 years	Cephalometric analysis of lateral cephalogram.	No significant changes were observed in cervical posture following the treatment with the Forsus appliance.
3.	Serritella E, Impellizzeri A, Musone L, De Stefano AA, Gabriella G	Cranio-cervical posture and rapid palatal expansion therapy [29] 2022	Rapid Expander of the Palate (REP), McNamara appliance	Cohort	35 subjects, between 6–14 years	Cephalometric analysis of lateral cephalogram.	No significant relationship between rapid palatal expansion therapy and changes in craniocervical posture.
4.	Kamal AT, Fida M	Evaluation of cervical spine posture after functional therapy with Twin-Block appliances: A retrospective cohort study [30] 2019	Twin-Block functional appliance	Cohort	60 subjects, exposed group was 11.8 ± 1.5 years, unexposed group was 11.6 ± 2.0 years.	Cephalometric analysis of lateral cephalogram.	Significant correlation between Twin-Block appliances, which leads to a more upright craniocervical posture.
5.	Venkatasubramanian P, Parameswaran, R., Vijayalakshmi, D	Quantitative analysis for the effect of orthodontic treatment on the body posture and its correlation with cervical posture in skeletal class II malocclusion—a clinical study [31] 2023	Orthodontic camouflage treatment	Cohort	18 subjects, over 18 years	Cephalometric analysis of lateral cephalogram in natural head position.	A statistically significant overextension of the head and increased spinal curvature following the orthodontic treatment.
6.	Ohnmeiß M, Kinzinger G, Wesselbaum J, Korbmacher-Steiner HM	Therapeutic effects of functional orthodontic appliances on cervical spine posture: a retrospective cephalometric study. [32] 2014	Activator, Bite-Jump Appliance (BJA) functional appliances	Cohort	64 subjects, mean age 11 years and 2 months	Cephalometric analysis of lateral cephalogram.	Significant upper cervical spine posture changes were observed after functional appliance treatment.

**Table 4:** Studies investigating craniocervical posture in the context of orthognathic surgery.

No.	Authors	Title Year	Orthognathic Surgery	Study Design	Sample Size and Ages	Posture Analysis Method	Conclusion
1.	Andriola FO, Kulczynski FZ, Deon PH, Melo DADS, Zanettini LMS, Pagnoncelli RM	Changes in cervical lordosis after orthognathic surgery in skeletal class III patients [33] 2018	Mandibular setback, Le Fort I osteotomy maxillary advancement.	Cohort	25 subjects, between 18–48 years	Cephalometric analysis of lateral cephalogram in natural head position and photographic method.	A significant cervical lordosis extension, alongside a non-significant head posture extension following mandibular setback.
2.	Cho D, Choi DS, Jang I, Cha BK	Changes in natural head position after orthognathic surgery in skeletal Class III patients [34] 2015	Mandibular setback.	Cohort	20 subjects, Class III intervention group (between 15.8–41.5 years) 20 subjects, Class I control group (between 16.7–37.3)	Cephalometric analysis of lateral cephalogram in natural head position and photographic method.	A significant change in natural head position, with a tendency towards head extension after mandibular setback.
3.	Lin X, Edwards SP.	Changes in natural head position in response to mandibular advancement [35] 2017	Mandibular advancement (±Genioplasty/ ±Le Fort I osteotomy).	Cohort	41 subjects, mean age 25 years	Cone-beam computed tomography	A significant correlation was found between the change in the mandibular position and a change in the head posture.
4.	Efendiyeva R, Aydemir H, Karasu H, Toygar-Memikoğlu U	Pharyngeal airway space, hyoid bone position, and head posture after bimaxillary orthognathic surgery in class III patients long-term evaluation [36] 2014	Mandibular setback, Le Fort I osteotomy.	Cohort	26 subjects, between 17 and 29 years	Cephalometric analysis of lateral cephalogram in natural head position.	No significant changes observed in head posture after orthognathic surgery.
5.	Kulczynski FZ, Andriola FO, Deon PH, Melo DADS, Pagnoncelli RM	Postural assessment in class III patients before and after orthognathic surgery [37] 2018	Mandibular setback, Le Fort I osteotomy.	Cohort	16 subjects, Class III intervention group (mean age: 30.81 ± 9.60) 15 subjects, Class III control group (mean age: 32.40 ± 15.74)	Photogrammetry	A significant posterior positioning of the head posture was observed after the orthognathic surgery.
